# Porous Nanocrystalline Silicon Supported Bimetallic Pd-Au Catalysts: Preparation, Characterization, and Direct Hydrogen Peroxide Synthesis

**DOI:** 10.3389/fchem.2018.00085

**Published:** 2018-03-27

**Authors:** Dmitriy I. Potemkin, Dmitry K. Maslov, Konstantin Loponov, Pavel V. Snytnikov, Yuri V. Shubin, Pavel E. Plyusnin, Dmitry A. Svintsitskiy, Vladimir A. Sobyanin, Alexei A. Lapkin

**Affiliations:** ^1^Laboratory of the Energy-Efficient Catalytic Processes, Novosibirsk State University, Novosibirsk, Russia; ^2^Department of Heterogeneous Catalysis, Boreskov Institute of Catalysis, Novosibirsk, Russia; ^3^Department of Chemical Engineering and Biotechnology, University of Cambridge, Cambridge, United Kingdom; ^4^Laboratory of the Rare Platinum Metals Chemistry, Nikolaev Institute of Inorganic Chemistry, Novosibirsk, Russia; ^5^Cambridge Centre for Advanced Research and Education in Singapore Ltd., Singapore, Singapore

**Keywords:** direct H_2_O_2_ synthesis, direct hydrogen peroxide synthesis, porous silicon, bimetallic nanoparticles, alloy nanoparticles, Pd-Au catalysts, gold-palladium catalysts, double complex salts

## Abstract

Bimetallic Pd-Au catalysts were prepared on the porous nanocrystalline silicon (PSi) for the first time. The catalysts were tested in the reaction of direct hydrogen peroxide synthesis and characterized by standard structural and chemical techniques. It was shown that the Pd-Au/PSi catalyst prepared from conventional H_2_[PdCl_4_] and H[AuCl_4_] precursors contains monometallic Pd and a range of different Pd-Au alloy nanoparticles over the oxidized PSi surface. The PdAu_2_/PSi catalyst prepared from the [Pd(NH_3_)_4_][AuCl_4_]_2_ double complex salt (DCS) single-source precursor predominantly contains bimetallic Pd-Au alloy nanoparticles. For both catalysts the surface of bimetallic nanoparticles is Pd-enriched and contains palladium in Pd^0^ and Pd^2+^ states. Among the catalysts studied, the PdAu_2_/PSi catalyst was the most active and selective in the direct H_2_O_2_ synthesis with H_2_O_2_ productivity of 0.5 ${\text{mol g}}_{\text{Pd}}^{-1}\;{\text{h}}^{-1}$ at selectivity of 50% and H_2_O_2_ concentration of 0.023 M in 0.03 M H_2_SO_4_-methanol solution after 5 h on stream at −10°C and atmospheric pressure. This performance is due to high activity in the H_2_O_2_ synthesis reaction and low activities in the undesirable H_2_O_2_ decomposition and hydrogenation reactions. Good performance of the PdAu_2_/PSi catalyst was associated with the major part of Pd in the catalyst being in the form of the bimetallic Pd-Au nanoparticles. Porous silicon was concluded to be a promising catalytic support for direct hydrogen peroxide synthesis due to its inertness with respect to undesirable side reactions, high thermal stability, and conductivity, possibility of safe operation at high temperatures and pressures and a well-established manufacturing process.

## Introduction

In the general field of industrial chemistry the role of heterogeneous catalysis is difficult to overestimate: the advances in large-scale catalytic processes, such as ammonia synthesis, sulphuric acid and nitric acid processes, ethylene and propylene production, acetic acid, and methanol syntheses and several others were responsible for the rapid development of human civilization in the twentieth-century. In the twenty-first century the challenges for catalysis are different but, arguably, even harder than those that were solved till now: selective activation of C-H bonds in saturated alkanes, efficient and selective activation of CO_2_, efficient splitting of water, highly selective functionalization of complex molecules for pharmaceutical and speciality chemicals, etc. These challenges have one common aspect—the requirement for much better control of reaction selectivity in the traditionally “difficult” reactions, such as sp3 C-H activation, as an example. The requirement for better selectivity control marks the need for a very different approach to catalysts design to that traditionally practiced in the twentieth-century for bulk processes: highly dispersed and uniform active sites are preferred (Flytzani-Stephanopoulos and Gates, 2012) and often polyfunctionality is required (Climent et al., 2014). Many conventional heterogeneous catalysts are highly *heterogeneous* in the nature of their active sites. For this reason, a significant effort in current research in the field of heterogeneous catalysis is on developing new methods of synthesis of heterogeneous catalysts, that result in much tighter control of the structure and composition of the active sites. Advanced synthesis techniques are under development: from modified impregnation and adsorption (Munnik et al., 2015) to a novel approach of nanoparticles biosynthesis which presents an alternative eco-friendly and potentially precise way for synthesis of heterogeneous catalysts (Hulkoti and Taranath, 2014).

Novel support materials do not frequently appear in catalytic literature and most conventionally-used supports, such as mixed oxides or carbon, are not well-suited to the challenge of precise control of selectivity due to their inherent heterogeneity. For this reason, significant attention is currently paid to “structured” supports, such as MOFs (Dhakshinamoorthy et al., 2013; Zhao et al., 2016), 2D materials such as graphene (Fan et al., 2015; Julkapli and Bagheri, 2015), 1D materials such as carbon nanotubes (Serp and Castillejos, 2010; Yan et al., 2015) or titanate nanotubes (Bavykin et al., 2005), and so on. Among the many support materials usually investigated in catalysis *silicon* is one of less known and little studied to date.

Its application as a catalyst support was first discussed in Polisski et al. (2010). PSi is a high surface area semiconducting material with high heat conductivity and pore structure very interesting for catalysis—a network of open pores with pore sizes in the mesopore range of 2–6 nm (Künzner et al., 2003); the electrochemical pore formation in silicon having been studied in detailed earlier (Lehmann et al., 2000). The high surface area of porous silicon (PSi) is hydrogen-terminated. This hydrogen can facilitate reduction of dissolved metal salts directly at the surface, without the use of additional reducing agents (Polisski et al., 2008). This can be exploited to produce small dispersed metal nanoparticles inside PSi pores from alcoholic metal salt solutions. This synthesis method resulted in metal nanoparticles in the range of 5–10 nm, located largely within the pores of the support. This is distinct from the earlier reports of metal/PSi composites obtained from aqueous metal salt solutions using metal hydrides or hydrogen as reducing agents, which result in the formation of much larger metal particles (Coulthard et al., 1993, 2000; Coulthard and Sham, 1998). Similarly, the surfactant mediated method using sodium borohydride as reducing agent in aqueous-ethanol solution results in the formation of large metal nanoparticles in the range of 10–50 nm (Yashtulov et al., 2011, 2016).

In recent years there is a significant increase in the number of publications devoted to different synthesis methods and application fields of metal-PSi materials. To cite only few studies, porous silicon was used to synthesize nano-composite materials for lithium-ion batteries (Zhai et al., 2017), as a stoichiometric reducing agent for direct conversion of CO_2_ to methanol (Dasog et al., 2017), as a support for Pd nanoparticles in catalytic oxidation of formic acid (Yashtulov and Flid, 2013), in a detailed experimental and theoretical investigation of the electronic structure and gas adsorption on Pd, Cu, Pd-Cu, and WO_3_-Pd nanoparticles supported on porous silicon for gas sensing applications (Litovchenko et al., 2017), for developing Pt-Pd/PSi electrocatalysts for direct methanol fuel cell (Ensafi et al., 2015). There are no new works on developing heterogeneous catalysts for chemical processes. Thus, in order to expand current knowledge on catalysis by porous silicon-supported catalysts we chose a difficult and very important industrial chemistry reaction of direct synthesis of hydrogen peroxide.

Hydrogen peroxide is an excellent oxidizing reagent for the production of both fine and bulk chemicals that finds applications also in the area of wastewater treatment, paper and pulp bleaching, and widely used in healthcare. Most of the world's hydrogen peroxide is currently produced by the sequential hydrogenation and oxidation of an alkyl anthraquinone (Bernardotto et al., 2009). This process suffers from several limitations, such as significant amount of organic waste, need of several energy consuming separation and concentration steps and economic feasibility only in large-scale (Menegazzo et al., 2015).

**Figure 1:**
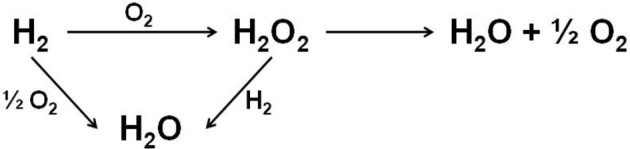
A general scheme of reactions involved in the direct H_2_O_2_ synthesis process.

The direct synthesis of H_2_O_2_ from hydrogen and oxygen in methanol or water reaction media provides a more atom efficient route to the current commercial production process. The direct route would enable H_2_O_2_ production to take place at its point of use, reducing the transportation costs, and for this reason there is a significant interest in developing such a catalytic process. The minimal required H_2_O_2_ concentration produced by the direct synthesis for practical use is estimated to be 1 wt.% or 0.23 M in methanol solution (Garcia-Serna et al., 2014). Figure 1 represents reactions involved in the direct H_2_O_2_ production process (Bernardotto et al., 2009). Besides the target H_2_O_2_ synthesis reaction, undesirable total H_2_ oxidation together with H_2_O_2_ hydrogenation and decomposition reactions could occur. Thus, catalyst's ability to synthesize H_2_O_2_ selectively and avoid its hydrogenation and decomposition becomes the key factor to reach high H_2_O_2_ concentrations with acceptable atomic and economic efficiencies.

Several reviews (Samanta, 2008; Dittmeyer et al., 2015; Edwards et al., 2015; Yi et al., 2016; Seo et al., 2017) consider catalytic systems, reaction conditions, and engineering concepts for direct H_2_O_2_ synthesis. Several bimetallic Pd-based systems show promising levels of performance in the direct H_2_O_2_ synthesis: Pd-Au (Edwards et al., 2005, 2009), Pd-Pt (Bernardotto et al., 2009), Pd-Ni (Maity and Eswaramoorthy, 2016), novel Pd-Te (Tian et al., 2017), and Pd-Ag (Gu et al., 2016) and the recently published Pd-Sn (Freakley et al., 2016), exhibiting a very high level of performance. Among other systems the Pd-Au one exhibiting selectivities up to 95% (Edwards et al., 2009) is the most extensively studied. Earlier studies have shown that bimetallic alloy Pd-Au nanoparticles, probably with Pd-enriched shell (Edwards et al., 2005), are responsible for high activity and selectivity in H_2_O_2_ synthesis. The surface ratio of Pd^0^/Pd^2+^ in the AuPd alloy was found to be an important factor controlling the overall catalyst performance. A stable non-leaching form of Pd^2+^ was identified as a key feature in highly selective PdAu catalysts (Edwards et al., 2012). Catalyst surface and/or reaction media acidity play important role in the reaction. Highly acidic cesium salts of Keggin-type tungstophosphoric acid (Cs_x_H_3−x_PW_12_O_40_) were proposed as the best support materials, providing better performance than carbon and metal oxides supports (Ntainjua et al., 2012). A reversible high-pressure CO_2_-derived acidification of combined water-methanol reaction media (Freakley et al., 2013) was successfully applied for high pressure operations. It should also be noted that in spite of huge efforts to determine the surface structure of bimetallic Pd-Au catalysts active for the H_2_O_2_ direct synthesis, the detailed understanding of the active sites of the metal nanoparticles remains elusive.

The selective formation of bimetallic Pd-Au nanoparticles in supported catalysts is desirable to obtain highly selective direct H_2_O_2_ synthesis catalyst, due to ability of monometallic Pd particles to catalyze hydrogenation of H_2_O_2_ (Edwards et al., 2015), thereby limiting the achievable H_2_O_2_ maximum concentration and decreasing process selectivity. Within this context we applied the approach based on decomposition of double complex salts (DCS) inside the pores of a support material for the synthesis of the bimetallic Pd-Au direct H_2_O_2_ synthesis catalyst. DCSs of transition metals, coordination compounds with the common formula [M′L′]_x_[M″L″]_y_, where M′ and M″ are metals, L′ and L″ are different ligands, have a number of advantages as single-source precursors of bimetallic catalysts:

- Complexing metals in a DCS are “mixed” on a molecular level that allows formation of alloys upon salt decomposition;
- Stoichiometry of a precursor complex determines composition of the alloys;
- Ability to preset exact phase composition and morphology of the alloy nanoparticles, varying the DCS deposition process, and decomposition conditions.

The successful application of DCS for catalyst preparation was demonstrated in a number of works (Potemkin et al., 2012, 2014, 2017; Shubin et al., 2012; Simonov et al., 2012; Bulushev et al., 2013; Vedyagin et al., 2013, 2014). To provide Pd-Au interaction and to promote alloy formation we have chosen [Pd(NH_3_)_4_][AuCl_4_]_2_ DCS as a single-source precursor. Its properties and application as a precursor for bimetallic Pd-Au nanoparticles were discussed in Shubin et al. (2012), Bulushev et al. (2013), Simonov et al. (2012), and Plyusnin et al. (2007). Due to the fact, that Pd and Au atoms are premixed in the precursor, [Pd(NH_3_)_4_][AuCl_4_]_2_ reduction inside the pore space at mild conditions leads to the selective formation of supported Pd-Au alloy nanoparticles without intermediate formation of Pd and Au phases and its alloying.

Besides the composition and structure of metallic component the choice of support plays an important role in designing direct H_2_O_2_ synthesis catalysts. Support's properties, such as red-ox interaction with Pd, wettability by reaction media, inertness with regard to H_2_O_2_ decomposition and hydrogenation, surface acidity are of special attention. Carbon supports (Edwards et al., 2009), titania (Edwards et al., 2005), Keggin-type heteropolyacids (Ntainjua et al., 2012), sulfated ceria and zirconia (Menegazzo et al., 2015) are considered as conventional choice for direct H_2_O_2_ synthesis catalysts. However, the search for alternative supports is still actual, especially in the scope of further reaction translation from lab-scale batch reactors to more practical and scalable continuous flow multiphase reactors, in which the application of conventional catalysts powders is limited by the need of its fixing and mass-/heat-transfer problem.

In this regard we investigated the properties of porous nanocrystalline silicon powder (PSi) as a support for Pd-Au direct H_2_O_2_ synthesis catalysts. We report results on preparation, characterization and catalytic tests of bimetallic PSi supported Pd-Au catalysts prepared by [Pd(NH_3_)_4_][AuCl_4_]_2_ single-source precursor decomposition. To our knowledge it is the first report of such catalytic system and extends the knowledge of porous silicon as a promising catalyst support. The comparison with monometallic PSi supported Pd and Au catalysts, and bimetallic Pd-Au catalyst, prepared utilizing conventional H_2_[PdCl_4_] and H[AuCl_4_] precursors, were done as well.

## Experimental

### Materials

Porous nanocrystalline silicon with average grain size of 4 μm was supplied by Vesta Ceramics. All other chemicals were commercially purchased and used without additional purification.

### Catalyst preparation

All catalysts were prepared by the metals reduction from its salts solutions by PSi's surface H-terminal atoms by the protocol adapted from Polisski et al. (2010).

The Pd loading in all catalysts was set to 2 wt.% which is close to optimal value according to literature (Edwards et al., 2005).

The synthesized [Pd(NH_3_)_4_][AuCl_4_]_2_ DCS was used as a single-source precursor for the PdAu_2_/PSi catalyst. The Pd and Au loadings were set to 2 and 7.4 wt.% according to [Pd(NH_3_)_4_][AuCl_4_]_2_ stoichiometry. A portion of 2 g of PSi powder was added to 50 mL of the DCS acetone solution of the required concentration at −15°C under vigorous stirring. Acetone solvent was used to attain wetting of the hydrophobic H-terminated surface of PSi and due to high [Pd(NH_3_)_4_][AuCl_4_]_2_ solubility in acetone. The suspension was kept at −15°C for 1 h under vigorous stirring, and then temperature was slowly increased to ambient. The obtained catalyst was filtered, washed several times with acetone and warm water to dissolve possible residual salts in pores, and dried at room temperature on air.

Bimetallic Pd-Au/PSi catalyst was prepared according to the procedure described above using the conventional H_2_[PdCl_4_] and H[AuCl_4_] joint water-ethanol solution as a metal source. The Pd and Au loadings for Pd-Au/PSi catalyst were set to 2 wt.%, which are close to the optimal values according to the literature data (Edwards et al., 2009).

Monometallic Pd/PSi and Au/PSi catalysts were prepared using the same protocol with water-acetone solutions of [Pd(NH_3_)_4_](NO_3_)_2_ and H[AuCl_4_] as precursors.

### Catalytic activity study

Catalytic tests were carried out at atmospheric pressure at −10°C in a thermostated glass reactor. Mixing was carried out with an inert rotor operating at 400 rpm. Oxygen and hydrogen were bubbled by a gas diffuser directly into the liquid phase with a total flow of 50 ml (STP) min^−1^. A non-explosive gas mixture with the following composition was used: 4 vol.% H_2_ and 96 vol.% O_2_. The reaction medium was 100 mL of a 0.03 M H_2_SO_4_ methanol solution. Catalyst loading of 50 mg was used. After the catalyst addition the suspension was stirred and then kept for 20 min in an ultrasonic bath. After that samples were pretreated *in situ* at room temperature first by H_2_ [30 min, 30 ml (STP) min^−1^] and then by O_2_ (20 min, 30 scm^3^ min^−1^). Prior to starting the reaction, reactor was purged with He [50 ml (STP) min^−1^]. During catalytic tests small aliquots of the liquid phase were sampled through a septum and used for hydrogen peroxide concentration determination.

H_2_O_2_ concentration was measured by iodometric titration. H_2_ concentrations at the inlet and the outlet of the reactor were measured by a quadrupole MS (Stanford research). The catalyst's performance was characterized in terms of the H_2_O_2_ concentration in methanol solutions ([H_2_O_2_]), H_2_ conversion (X_H2_) and the selectivity toward H_2_O_2_ synthesis (S_H2O2_), which were calculated from the following equations:

$${\text{X}}_{H_{2}}=\frac{{\left[{\text{H}}_{2}\right]}_{\text{inlet}}-{\left[H_{2}\right]}_{outlet}}{{\left[{\text{H}}_{2}\right]}_{\text{inlet}}}\cdot 100\%,$$

$${\text{S}}_{H_{2}O_{2}}=\frac{{\text{n}}_{{\text{H}}_{2}{\text{O}}_{2}}}{\Delta n_{H_{2}}}\cdot 100\%,$$

where [H_2_]_inlet_ and [H_2_]_outlet_ are the average H_2_ inlet and outlet concentrations during the period between aliquots sampling; n_H_2_O_2__is the amount of H_2_O_2_ formed during the period between aliquots sampling; Δn_H_2__is the amount of H_2_ consumed during the period between aliquots sampling.

The catalyst loading and reaction temperature were optimized in order to make hydrogen conversion insensitive to stirring speed.

Experiments on H_2_O_2_ decomposition and hydrogenation were carried out at the same conditions as H_2_O_2_ synthesis. The initial H_2_O_2_ concentration was set to *ca*. 70 mM (70·10^−3^ mol L^−1^), gas feed composition: pure He for H_2_O_2_ decomposition and 4 vol.% H_2_ and 96 vol.% He for H_2_O_2_ hydrogenation tests. The gas flow was set to 50 scm^3^ min^−1^. H_2_O_2_ concentration was measured one time per hour during 5 h.

Pure PSi support was treated by the water-acetone solution using the protocol described in section Catalyst Preparation and was tested in H_2_O_2_ synthesis, decomposition and hydrogenation. PSi did not show any activity in this reaction. Thus, one can conclude that PSi material is inert with regard to H_2_O_2_ synthesis, decomposition, and hydrogenation and could be successfully used as a catalyst support for direct synthesis of H_2_O_2_.

### Catalyst characterization

The as-prepared catalysts were characterized by a number of physico-chemical techniques. The chemical composition of the catalysts was determined by inductively coupled plasma atomic emission spectrometry (an Optima instrument).

The specific BET surface area (S_BET_) and pore volume (V_p_) of the catalysts were determined from the complete nitrogen adsorption isotherms at −196°C (ASAP 2400 instrument).

XRD analysis of the as-synthesized sample powders was performed on a “DRON-SEIFERT-RM4” diffractometer (Cu*K*_α_ irradiation, graphite monochromator *d*_001_ = 3.345 Ǻ, room temperature). The scanning range was 20°−55° (2θ) with a step of 0.1°. The experimental diffraction data were processed using the PowderCell v.2.4 (Kraus and Nolze, 2000) and WINFIT 1.2.1 (Krumm, 1996) programs, which allowed a calculation of the quantitative phase composition, the lattice parameters, and the crystallite size (D_XRD_). Data from the JCPDS-ICDD database (Powder Diffraction File, 2009) were used as reference. The composition of the Au_x_Pd_1−x_ alloys was determined using the experimentally measured dependence between lattice parameters of face-centered cubic cell (fcc, space group Fm-3m) in gold-palladium solid solutions and the atomic fraction of constituent elements (Shubin et al., 2012).

Transmission electron microscopy (TEM) study was performed using a JEM-2010 (lattice plane resolution 0.14 nm at accelerating voltage of 200 kV) equipped with an energy-dispersive X-ray (EDX) spectrometer EDAX [Si(Li) detector with 130 eV energy resolution]. The samples for the TEM study were prepared on perforated carbon film mounted on a copper grid.

Fourier transform infrared spectroscopy (FTIR) analysis of the catalyst samples was performed using Shimadzu IRAffinity spectrometer using KBr as a diluent.

X-ray photoelectron spectroscopic (XPS) analysis was performed using an ES-300 (KRATOS Analytical) photoelectron spectrometer. MgKα (hν = 1253.6 eV) X-ray source was applied for spectra registration. The spectrometer calibration was performed using bulk gold (Au4f_7/2_) and copper (Cu2p_3/2_) photoelectron lines with binding energy (BE) values of 84.0 and 932.7 eV, respectively. The position of the Si2p line for oxidized silicon with BE = 103.3 eV was used as reference for spectra calibration. Due to the overlapping of Au4d- and Pd3d- spectral regions (Smolentseva et al., 2011), the subtraction of Au4d line from Au/PSi sample was applied for all bimetallic catalysts to analyze palladium surface species precisely. The chemical composition of the surface was quantitatively determined from integral peak areas using standard atomic sensitivity factors (ASFs) (Wagner et al., 2013). Processing of the obtained data and spectral analyses were performed using homemade XPS-Calc program, which has been tested on a number of systems (Svintsitskiy et al., 2013). The curve fitting procedure was performed using an approximation based on a combination of the Gaussian and Lorentzian functions with the subtraction of a Shirley-type background. Before the curve fitting, all spectra were smoothed using a Fourier filter. No considerable difference between the smoothed and experimental curves was observed (standard deviation <1%).

## Results

### Catalysts characterization

Table 1 shows actual metal loading, specific surface area and pore volume of the prepared samples. The metal loading was close to the calculated value for all the samples studied. S_BET_ changed from 154 m^2^ g^−1^ for the pure PSi to 104 m^2^ g^−1^ for the PdAu_2_/PSi, respectively. V_p_ decreased synchronously with S_BET_ from 0.25 cm^3^ g^−1^ for the pure PSi to 0.17 cm^3^ g^−1^ for the PdAu_2_/PSi.

**Table 1:** Catalyst's metal loading, specific BET surface area and pore volume.

**Catalyst**	**Precursor**	**S_BET_, m^2^/g**	**V_p_, cm^3^/g**	**Pd, wt.%**	**Au, wt.%**	**Pd/(Pd+Au) atomic ratio**
PSi	–	154	0.25	0	0	–
Au/PSi	H[AuCl_4_]	133	0.20	0	5	1
Pd/PSi	[Pd(NH_3_)_4_](NO_3_)_2_	141	0.20	2.1	0	0
Pd-Au/PSi	H_2_[PdCl_4_]+H[AuCl_4_]	127	0.19	2.1	2	0.66
PdAu_2_/PSi	[Pd(NH_3_)_4_][AuCl_4_]_2_	104	0.17	1.9	7.3	0.33

**Figure 2:**
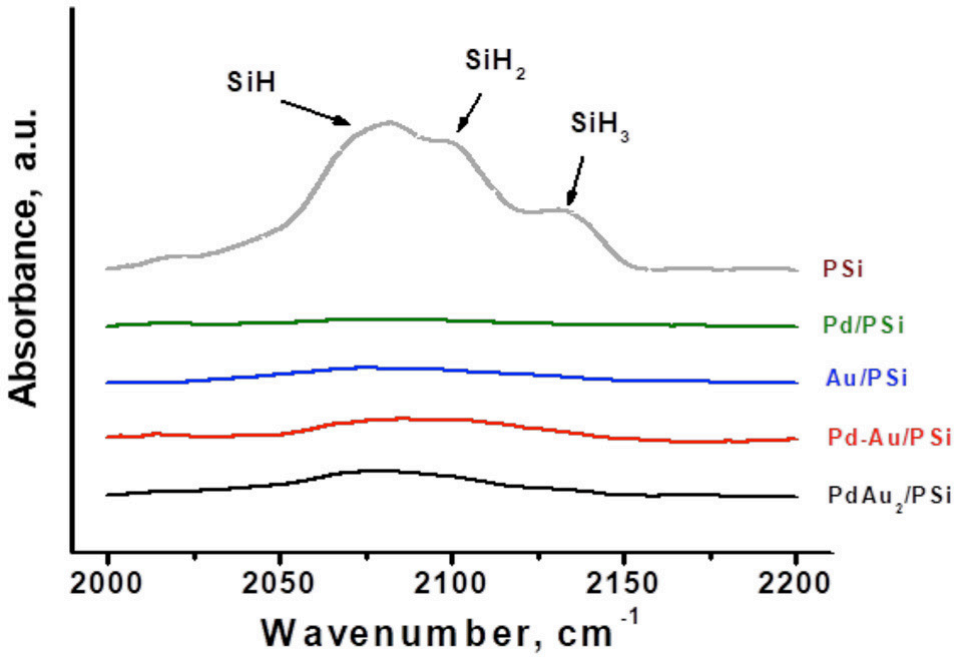
The IR-spectra of PSi support and Pd/PSi, Au/PSi, Pd-Au/PSi, and PdAu_2_/PSi catalysts.

PSi contains surface H-terminal atoms, which can act as an internal surface reducing agent facilitating metal reduction. The presence of such H-terminal atoms in fresh (as received samples stored on air) non-oxidized PSi support and catalyst samples was monitored by FTIR. Figure 2 shows IR-spectra of the PSi support before catalyst preparation and of Pd/PSi, Au/PSi, Pd-Au/PSi, and PdAu_2_/PSi catalysts. The PSi spectrum contains the stretching modes of Si-H_x_ bonds (2,082, 2,107, and 2,132 cm^−1^ for *x* = 1, 2, and 3, respectively) (Ogata et al., 2000; Polisski et al., 2010). The IR-spectra of the catalysts do not show stretching modes of Si-H_x_ bonds, indicating that all H-terminal atoms were spent during catalysts preparation.

**Figure 3:**
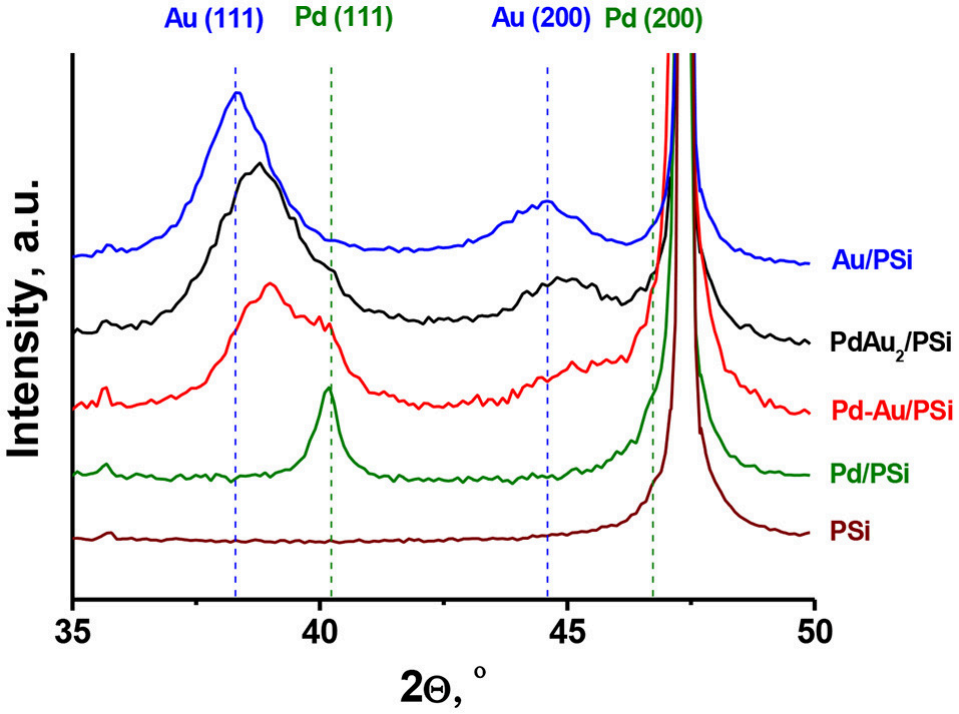
XRD patterns of PSi support and Pd/PSi, Au/PSi, Pd-Au/PSi, and PdAu_2_/PSi catalysts.

Figure 3 shows XRD patterns of the catalysts studied and of the PSi support. The silicon phase (2Θ = 47.30°) was identified for all the samples. For the Pd/PSi and the Au/PSi catalysts, metallic Pd (2Θ = 40.15° and 46.70°) with the crystallite size (D_XRD_) of 12 nm and Au (2Θ = 38.32° and 44.62°) with the crystallite size of 5 nm, respectively, were identified, see also Table 2. The XRD pattern of Pd-Au/PSi contains not fully resolved peaks at 2Θ = 40.08° and 39.02°, which could be assigned to metallic Pd (D_XRD_ = 6.3 nm) and solid solution Pd_0.35_Au_0.65_ (D_XRD_ = 5.5 nm). Bimetallic nanoparticles are gold-enriched compared to the data on elemental composition, Table 1, due to the presence of significant amount of monometallic Pd nanoparticles. The XRD pattern of PdAu_2_/PSi catalyst contains broad peaks at 2Θ = 38.79° s 45.01°, which could be assigned to the solid solution Pd_0.25_Au_0.75_ nanoparticles with the crystallite size of 4 nm. The composition of Pd_0.25_Au_0.75_ solid solution is relatively close to the stoichiometry of DCS and data on elemental composition, Table 1. Most likely, small enrichment by gold is caused by the presence of pure Pd particles, which is confirmed by the presence of a shoulder at the position of metallic Pd, Figure 3. The obtained data allows us to estimate that *ca*. 2/3^s^ of Pd in the PdAu_2_/PSi catalyst is present in the form of bimetallic particles, while 1/3rd is present in the form of pure Pd. Assuming the similar particle size and taking into account the significant dilution by gold in the bimetallic particles the quantitative ratio of bimetallic to pure Pd particles in the catalyst could be estimated to be not <8, thus indicating that the majority of particles are bimetallic.

**Table 2:** Catalysts phase composition, relating to Figure 3.

**Catalyst**	**Phase composition**	**Crystallite size (D_XRD_), nm**
Au/PSi	Au	5
Pd/PSi	Pd	12
Pd-Au/PSi	Pd_0.35_Au_0.65_	5.5
	Pd	6.3
PdAu_2_/PSi	Pd_0.25_Au_0.75_	4

**Figure 4:**
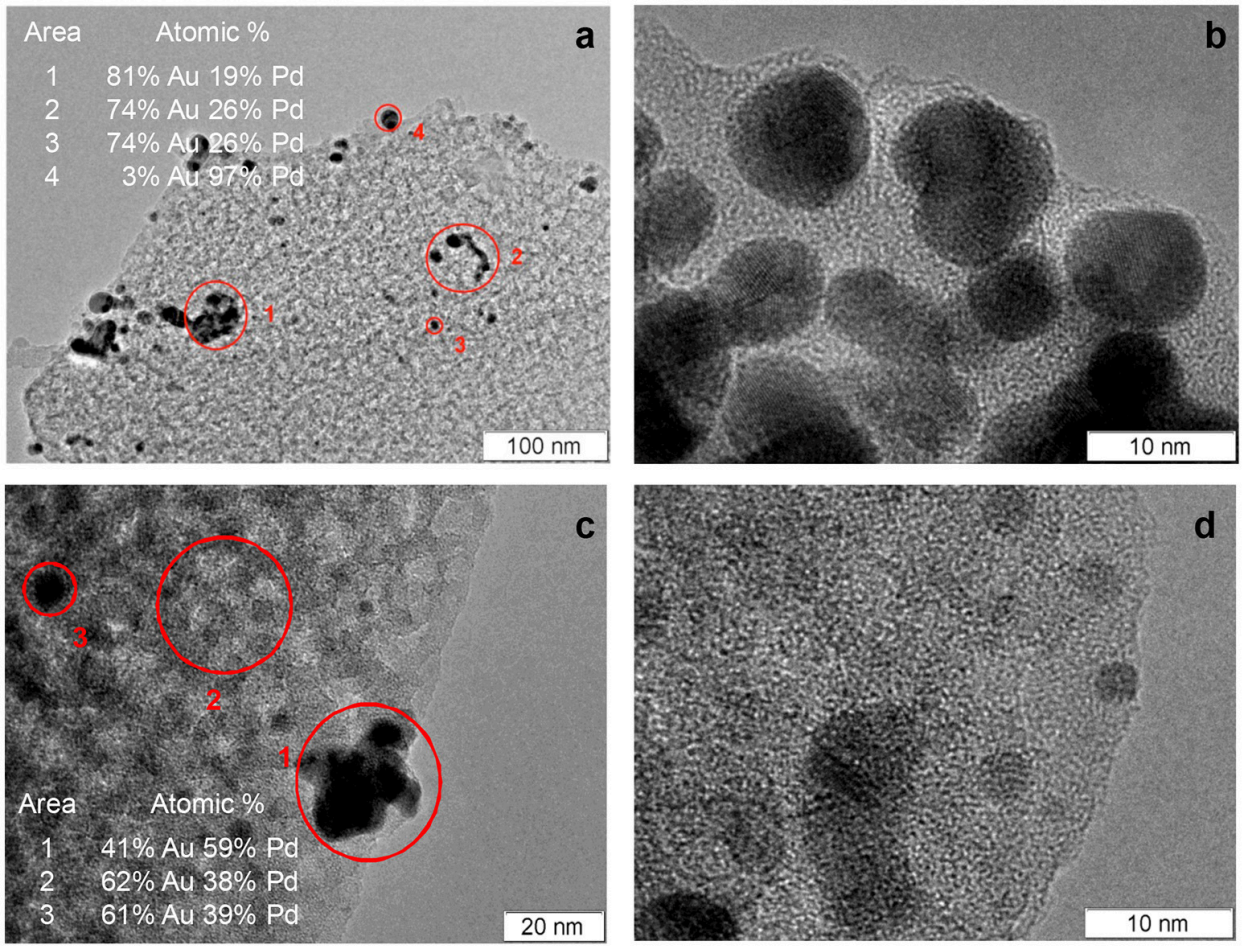
TEM images of PdAu_2_/PSi **(a**,**b)** and Pd-Au/PSi **(c**,**d)** catalysts and EDX analysis data **(a**,**c)**.

PdAu_2_/PSi and Pd-Au/PSi catalysts were studied by TEM and EDX. PdAu_2_/PSi contains agglomerates of bimetallic nanoparticles with the sizes in the range of 5–10 nm, see Figures 4a,b. According to the EDX data, composition of the nanoparticles is non-uniform: some particles are enriched by Pd. However, the average composition of agglomerates is close to Pd_0.25_Au_0.75_, that was also identified by the XRD. Figures 4c,d show that Pd-Au/PSi also contains the agglomerates of bimetallic nanoparticles with the sizes in the range of 3–20 nm. The composition of nanoparticles is non-uniform and varies from pure Pd to Au-enriched alloys. The peaks of Pd-Au alloys at XRD patterns of PdAu_2_/PSi and Pd-Au/PSi catalysts have a certain degree of asymmetry, indicating the non-uniformity of alloy nanoparticles composition, in a good agreement with EDX data. The analysis of distribution of particles composition and complex XRD fitting requires much wider array of TEM and EDX data and is out of the scope of this work. However, the majority of metallic particles in Pd-Au/PSi and practically all in PdAu_2_/PSi catalysts are bimetallic.

**Figure 5:**
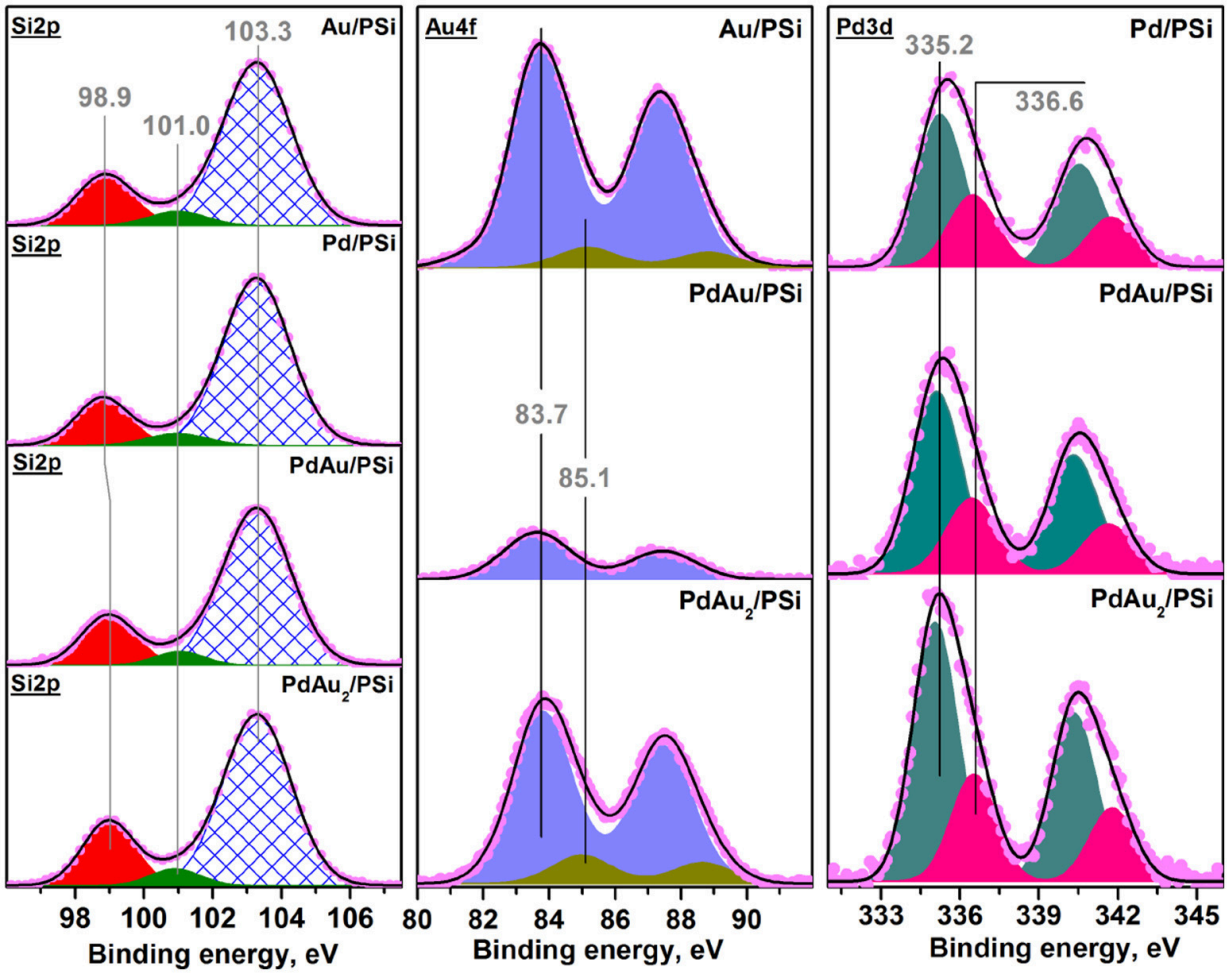
Photoelectron Si2p-, Au4f-, and Pd3d- XP spectra of Pd/PSi, Au/PSi, Pd-Au/PSi, and PdAu_2_/PSi catalysts.

Figure 5 shows the X-ray photoelectron spectra of the bimetallic Pd-Au catalysts in comparison with the monometallic Pd/PSi and Au/PSi samples. Spectral Si2p-regions were the same for all studied samples and consisted of three components with BE(Si2p) of ~99, 101 and 103.3 eV. According to the literature data, Si2p peaks with BE ~99 and 103.3 eV can be attributed to Si^0^ and oxidized Si^4+^ (SiO_2_-type) species, respectively (Wagner et al., 2013). The position of oxidized Si^4+^ peak was found to be stable during spectra registration and was chosen as a reference for spectra calibration. The Si2p peak with BE ~101 eV was characterized by the lowest intensity and could be interpreted as an interface SiO_x_ between silicon and surface oxidized SiO_2_-type layer. The quantitative ratio between different silicon states is given in Table 3. Therefore, surface of porous silicon used as a support material was found to be partially oxidized, which is expected for PSi materials not stored in an oxygen-free environment.

**Table 3:** XPS data on the surface composition of the catalysts.

**Catalyst**	**Pd/PSi**	**Au/PSi**	**Pd-Au/PSi**	**PdAu_2_/PSi**
(Pd/Si)·10^2^	1.3	–	1.1	1.4
(Au/Si)·10^2^	–	3.1	0.6	2.5
Pd/Au	–	–	1.8	0.6
Pd^2+^, %	31	–	27	29
Au^δ+^, %	–	8	–	13
Si^0^, %	18	20	19	22
SiO_x_, %	5	6	6	5
Si^4+^, %	77	75	75	73

Quantitative XPS analysis has also shown that surface Pd amount was similar for bimetallic and monometallic samples (Pd/Si~0.013), while gold amount was found to be lower for bimetallic catalyst (Au/Si~0.006 and 0.0025) than for Au/PSi (Au/Si~0.031). This indicates enrichment of the surface of the bimetallic samples by palladium. Surface palladium enrichment can be caused by interaction with oxygen followed by formation of the oxidized palladium species (Hilaire et al., 1981). As seen from Figure 5, two doublet components with BE ~335.2 and 336.6 eV identified as Pd3d_5/2_ were observed in Pd3d spectra for all studied samples. The doublet with BE ~335.2 eV can be attributed to metallic palladium, while the second Pd3d component is caused by the presence of oxidized Pd^2+^ state, probably, in PdO form (Smolentseva et al., 2011). Hence, the observed segregation of palladium can be related to the process of surface oxidation. The amount of Pd^2+^ was similar for all the studied catalysts and equal to *ca*. 30%, see Table 3.

Detailed XPS analysis of the Au-containing samples showed presence of two Au4f doublet components with BE ~83.7 and 85.1 eV (Au4f_7/2_). These were attributed to metallic Au^0^ and weakly oxidized Au^δ+^ species, respectively (Ballestero et al., 2015).

Results of the analysis of the catalysts' surface composition reveal that the surface of the bimetallic Pd-Au nanoparticles is enriched by Pd atoms present in Pd^0^ and Pd^2+^ states. It should be noted that according to literature (Ouyang et al., 2015) the presence of both, Pd^0^ and PdO, forms on the catalysts surface is required for the catalysts to be active in direct synthesis of H_2_O_2_.

One can conclude that the Pd-Au/PSi catalyst prepared by the conventional H_2_[PdCl_4_] and H[AuCl_4_] precursors contains monometallic Pd and a wide range of different Pd-Au solid solution nanoparticles over the oxidized PSi surface, whereas the PdAu_2_/PSi catalyst prepared by [Pd(NH_3_)_4_][AuCl_4_]_2_ DCS single-source precursor reduction predominantly contains bimetallic Pd-Au solid solution nanoparticles with the composition close to Pd_0.25_Au_0.75_. For both catalysts surface of bimetallic nanoparticles is Pd-enriched and contains palladium in Pd^0^ and Pd^2+^ states.

### H_2_O_2_ direct synthesis

The PSi supported catalysts were tested in H_2_O_2_ direct synthesis at T = −10°C and P = 1 bar in a batch reactor with bubbling H_2_-O_2_ gas mixture and 0.03 M H_2_SO_4_ methanol solution as a reaction media. Methanol could be suggested as one of the best solvents for this reaction due to high H_2_ and O_2_ solubilities, inability to form dangerous organic peroxides and the fact that it is a favorable solvent for many oxidation reactions involving H_2_O_2_ (Melada et al., 2006), such as propylene epoxidation process (Biasi et al., 2013).

After each catalytic test the catalyst powder was filtered and the residual reaction media was tested in the reaction conditions for 1 h in order to check metal leaching from the catalysts. None of the filtrates showed any activity in H_2_O_2_ synthesis or decomposition, indicating that PSi-supported metallic nanoparticles were stable under the reaction conditions tested, or leached metals were inactive in any relevant catalytic reactions. Filtered catalyst powders were recycled to the 2nd 3 h long catalytic run and properties of all catalysts were fully reproduced with respect to amount of catalyst recycled.

**Figure 6:**
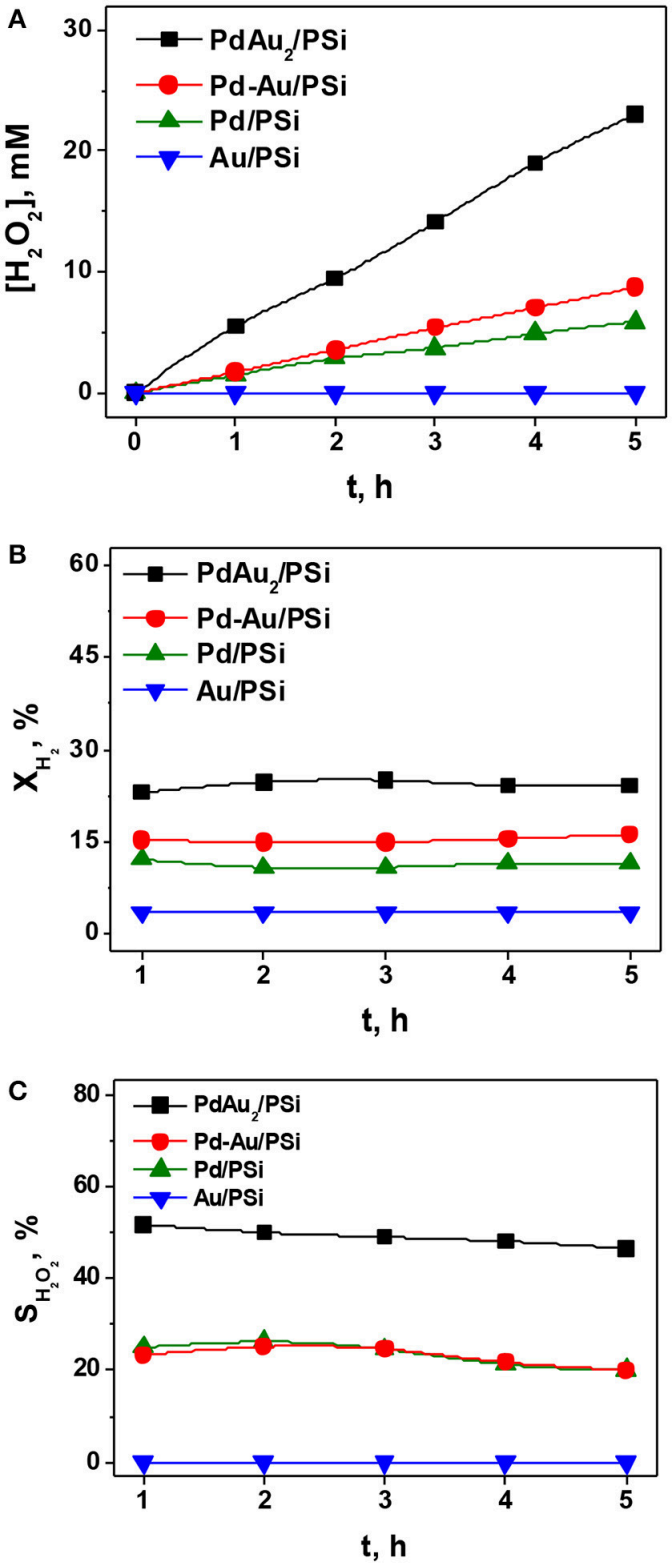
The time course of the H_2_O_2_ concentration **(A)**, H_2_ conversion **(B)**, and H_2_O_2_ selectivity **(C)** for the direct H_2_O_2_ synthesis over the Pd/PSi, Au/PSi, Pd-Au/PSi, and PdAu_2_/PSi catalysts. P = 1 bar. T = −10°C. Reaction media: 100 mL of a 0.03 M H_2_SO_4_ methanol solution. Gas flow rate: 2 ml (STP) min^−1^ H_2_ and 48 ml (STP) min^−1^ O_2_. Catalyst loading: 50 mg.

Figure 6 shows the time course of H_2_O_2_ concentration (Figure 6A), H_2_ conversion (Figure 6B) and selectivity (Figure 6C) for the direct H_2_O_2_ synthesis over Pd/PSi, Au/PSi, Pd-Au/PSi, and PdAu_2_/PSi catalysts. It is seen that the Au/PSi catalyst was inactive in the direct H_2_O_2_ synthesis as well as in complete H_2_ oxidation: H_2_ conversion was <4%. The Pd-containing catalysts were active in the direct H_2_O_2_ synthesis. For all the Pd-based catalysts H_2_O_2_ concentration grows nearly linearly with time, H_2_ conversion is constant, while selectivity slowly decreases. The H_2_O_2_ concentration of *ca*. 6 mM was reached over Pd/PSi after 5 h on stream at X_H2_ of 10% and S_H2O2_ decrease from 25 to 20%. The Pd-Au/PSi catalyst was more active: [H_2_O_2_] of *ca*. 9 mM was reached after 5 h on stream at X_H2_ of 15% and S_H2O2_ decrease from 25 to 20%. The Pd/PSi and Pd-Au/PSi catalysts exhibited similar selectivity vs. time dependencies. This could be associated with the presence of monometallic Pd nanoparticles in both catalysts. Higher activity of Pd-Au/PSi could be associated with higher metal dispersion.

The PdAu_2_/PSi catalyst exhibited the best performance in the direct H_2_O_2_ synthesis. H_2_O_2_ concentration of *ca*. 23 mM was reached after 5 h on stream at X_H2_ of 23% and S_H2O2_ decreases from *ca*. 50 to *ca*. 45%. High activity of PdAu_2_/PSi in H_2_ oxidation could be associated with high metal dispersion (4 nm for PdAu_2_/PSi vs. 5–6 nm for Pd-Au/PSi vs. 12 nm for Pd/PSi), while high selectivity toward H_2_O_2_ synthesis could be associated with the major part of Pd in the catalyst being in the form of bimetallic Pd-Au nanoparticles, since monometallic Pd nanoparticles are known to be active in the undesirable H_2_O_2_ hydrogenation reaction. The obtained data on H_2_O_2_ direct synthesis shows that bimetallic PSi-supported catalysts are more active and selective (in the case of PdAu_2_/PSi), compared with the monometallic Pd/PSi, which is not surprising (Edwards et al., 2015; Li and Yoshizawa, 2015).

It was shown, that for Pd/PSi, Pd-Au/PSi, and PdAu_2_/PSi catalysts selectivity of H_2_O_2_ synthesis slowly decreased with time, while H_2_ conversion remained constant (Figures 6B,C). It could be caused by the occurrence of the undesirable H_2_O_2_ decomposition and hydrogenation reactions. In order to elucidate the influence of these reactions the H_2_O_2_ decomposition and hydrogenation reactions were studied over PSi-supported catalysts.

### H_2_O_2_ decomposition and hydrogenation

In order to study stability of the synthesized H_2_O_2_ at the reaction conditions a number of experiments on H_2_O_2_ decomposition and hydrogenation were carried out. Experimental conditions were close to that for H_2_O_2_ synthesis in this work. As initial reaction media 0.07 M H_2_O_2_/0.03 M H_2_SO_4_ methanol solution was chosen. The reactions were carried out at −10°C.

**Figure 7:**
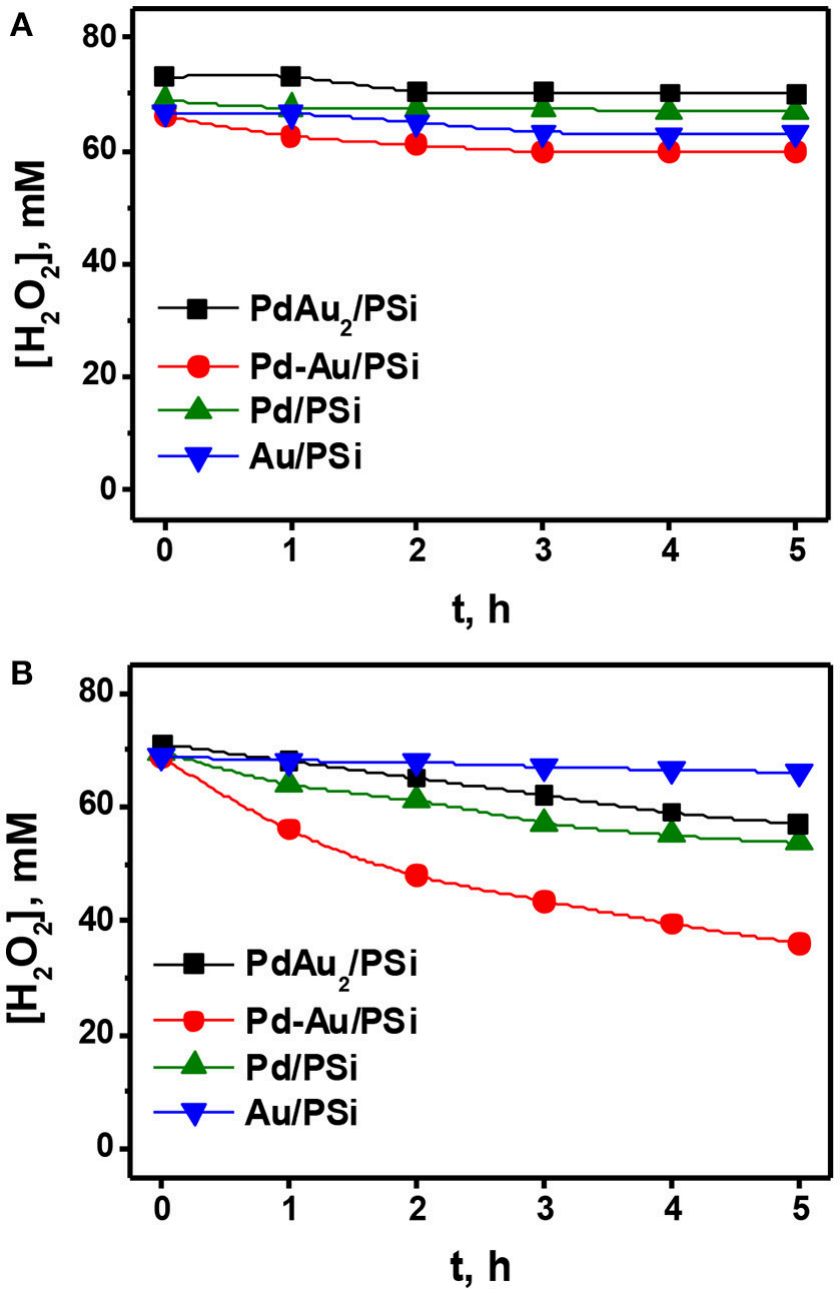
The time course of the H_2_O_2_ concentration for the H_2_O_2_ decomposition **(A)** and reduction **(B)** over the Pd/PSi, Au/PSi, Pd-Au/PSi and PdAu_2_/PSi catalysts. P = 1 bar. T = −10°C. Reaction media: 100 mL of a *ca*. 70 mM/0.03 M H_2_SO_4_ methanol solution. Gas flow rate: **(A)** 50 ml (STP) min^−1^ He; **(B)** 2 ml (STP) min^−1^ H_2_; and 48 ml (STP) min^−1^ He. Catalyst loading: 50 mg.

At first, the reaction of H_2_O_2_ decomposition was studied. Figure 7A shows the time course of H_2_O_2_ concentration for H_2_O_2_ decomposition over Pd/PSi, Au/PSi, Pd-Au/PSi, and PdAu_2_/PSi catalysts. It is seen, that H_2_O_2_ concentration was constant for the Pd/PSi, Au/PSi, and PdAu_2_/PSi catalysts and negligibly decreased in the case of Pd-Au/PSi. This indicates that all studied catalysts were generally inactive in H_2_O_2_ decomposition.

Figure 7B shows the results on H_2_O_2_ hydrogenation over the Pd/PSi, Au/PSi, Pd-Au/PSi, and PdAu_2_/PSi catalysts. It is seen, that H_2_O_2_ concentration was practically constant during 5 h over Au/PSi, indicating that Au/PSi was practically inactive in H_2_O_2_ hydrogenation. Over Pd/PSi and PdAu_2_/PSi catalysts H_2_O_2_ concentration slowly decreased from 70 mM to *ca*. 55 mM after 5 h on stream. Pd-Au/PSi was the most active one in H_2_O_2_ hydrogenation: H_2_O_2_ concentration decreased from 70 to 36 mM after 5 h on stream. The differences in behavior of the Pd-based catalysts are caused by the different phase compositions and dispersions of the active component. The PdAu_2_/PSi catalyst was less active in H_2_O_2_ hydrogenation than Pd-Au/PSi and Pd/PSi despite the higher metal dispersion due to the presence of the major part of Pd in the catalyst in the form of bimetallic Pd-Au nanoparticles.

Thus, one can conclude that, unlike H_2_O_2_ decomposition, H_2_O_2_ hydrogenation significantly influences direct H_2_O_2_ synthesis over the Pd/PSi, Pd-Au/PSi and PdAu_2_/PSi catalysts, decreasing reaction selectivity and limiting the maximum reachable H_2_O_2_ concentration. Among the catalysts studied the PdAu_2_/PSi is the least active in H_2_O_2_ hydrogenation.

## Discussion

Among the catalysts studied in the present work the PdAu_2_/PSi one showed the highest activity and selectivity in the direct synthesis of H_2_O_2_. This is due to its high activity in H_2_O_2_ synthesis reaction as well as low activity in the undesirable H_2_O_2_ decomposition and hydrogenation side reactions. We assign excellent performance of the PdAu_2_/PSi catalyst to the fact that most of Pd in the catalyst is in the form of bimetallic Pd-Au nanoparticles. The preferred method of synthesis of supported bimetallic Pd-Au nanoparticles was found to be based on DCS [Pd(NH_3_)_4_][AuCl_4_]_2_ reduction inside the PSi's pore space by the surface H-terminal atoms. The alloy nanoparticles are the primary product of DCS reduction. Prevention of intermediate formation of monometallic Pd and Au grains allows one to obtain bimetallic nanoparticles under very mild conditions. The composition of the formed Au_0.75_Pd_0.25_ nanoparticles is relatively close to the DCS stoichiometry. Thus, it could be concluded, that the presence of specific chemical interaction between the Pd and Au precursors promotes formation of supported bimetallic nanoparticles and, therefore, active and selective catalyst, while the application of conventionally used joint water solutions of Pd and Au chlorides leads to the formation of a wide range of different Pd-Au bimetallic nanoparticles as well as pure Pd and Au ones (e.g., the case of Pd-Au/PSi) (Edwards et al., 2009).

Despite the fact that surface of as received PSi was H-terminated, the surface of as-prepared catalysts was mostly covered by a SiO_2_ layer. From one side it presents the possibility for strong metal-support interaction (SMSI) in the Pd_x_Au_1−x_-PSi system, which is generally favorable for H_2_O_2_ direct synthesis catalysts due to electron transfer from Pd (Yi et al., 2016). However, the SMSI effect in the Si-based systems is typically observed after high temperature reduction and is associated with silicide phases or layers formation (Ueckert et al., 1997). This is not the case here. From the other side SiO_2_ is an acidic support with low value of surface isoelectric point of about 2, which is desirable for stabilization of the formed H_2_O_2_. Also, the SiO_2_ layer encapsulates the possible undesirable contaminations and provides the material inertness with respect to H_2_O_2_ decomposition and hydrogenation reactions.

Gemo et al. (2015) and Ouyang et al. (2015) highlighted the key role of Pd^2+^/Pd^0^ surface ratio on the Pd catalysts performance in the direct synthesis of H_2_O_2_. Simultaneous presence of Pd^0^ and Pd^2+^ sites at the surface is required to provide dissociative H_2_ adsorption and non-dissociative O_2_ adsorption. The single-metal Pd catalysts tend to oxidation under reaction conditions that is undesirable (Gemo et al., 2015). High values of ${\text{H}}_{2}^{\text{L}}$/Pd ratio (${\text{H}}_{2}^{\text{L}}$–the concentration of hydrogen dissolved in liquid media) and Pd particle size increase lead to the stabilization of Pd^0^ surface sites. We believe that Au addition to Pd together with selective formation of Pd-Au bimetallic particles increases its resistance toward oxidation as Au is a “very noble” metal. The Pd-Au particle size in the PdAu_2_/PSi catalyst is not very small. All these factors protect Pd surface from deep oxidation under reaction conditions and help to provide the acceptable Pd^2+^/Pd^0^ surface ratio and reach higher H_2_O_2_ selectivity.

The properties of PdAu_2_/PSi in direct H_2_O_2_ synthesis were compared with other Pd-based catalytic systems. Table 4 summarizes the literature data on catalytic performance and reaction conditions. PdAu_2_/PSi catalyst studied in the present work showed the H_2_O_2_ productivity of 0.5 $\text{mol }{\text{g}}_{\text{Pd}}^{-1}\;{\text{h}}^{-1}$ or 12.3 $\text{mol }{\text{kg}}_{\text{cat}}^{-1}\;{\text{h}}^{-1}$ at selectivity of 50%, that is lower than the productivity of the best-known catalysts. The lower H_2_O_2_ production rate could be associated with low reaction temperature of −10°C. The highest values of productivity were obtained for Pd_0.1_Au_0.0333_Cs_2.5_H_0.2_PW_12_O_40_ (29.4 $\text{mol }{\text{g}}_{\text{Pd}}^{-1}\;{\text{h}}^{-1}$) (Ntainjua et al., 2012) and Pd-Au/C (6.4 $\text{mol }{\text{g}}_{\text{Pd}}^{-1}\;{\text{h}}^{-1}$) (Edwards et al., 2009) catalysts. However, these studies utilized elevated pressures of 40 and 37 atm, respectively. In the works Bernardotto et al. (2009) and Ouyang et al. (2015), the experiments were carried at atmospheric pressure and the productivity values of 3.0 $\text{mol }{\text{g}}_{\text{Pd}}^{-1}\;{\text{h}}^{-1}$ for Pd/TiO_2_ (Svintsitskiy et al., 2013) and 1.0 $\text{mol }{\text{g}}_{\text{Pd}}^{-1}\;{\text{h}}^{-1}$ for PdAu_2_/ZrO_2_ (Bernardotto et al., 2009) were obtained. These are closer to the results obtained for the PdAu_2_/PSi catalyst, taking into account the higher reaction temperatures: 10°C for Pd/TiO_2_ and 25°C for PdAu_2_/ZrO_2_. Also, similar selectivities were obtained: 60% for Pd/TiO_2_ and PdAu_2_/ZrO_2_ vs. 50% for PdAu_2_/PSi.

**Table 4:** The comparison of catalyst performance and reaction conditions for direct H_2_O_2_ synthesis over different Pd-based systems.

**Catalyst**	**T,°C**	**P, bar**	**Gas composition**	**Solvent**	**S_H_2_O_2__, %**	**Productivity, $\frac{{\text{mol}}_{{\text{H}}_{\text{2}}{\text{O}}_{\text{2}}}}{{\text{kg}}_{\text{cat}}\cdot \text{h}}$**	**Productivity, $\frac{{\text{mol}}_{{\text{H}}_{\text{2}}{\text{O}}_{\text{2}}}}{{\text{g}}_{\text{Pd}}\cdot \text{h}}$**	**Maximum reached [H_2_O_2_], wt.%**	**References**.
PdAu_2_/PSi	−10	1	96 O_2_, 4 H_2_	H_2_SO_4_, CH_3_OH	50	12.3	0.5	0.1^a^	This work
Pd/TiO_2_	10	1	60 O_2_, 15H_2_, 25 N_2_	H_2_SO_4_, C_2_H_5_OH	60	29.9	3.0	–	Ouyang et al., 2015
PdAu_2_/ZrO_2_	25	1	96 O_2_, 4 H_2_	H_2_SO_4_, CH_3_OH	60	13.7	1	0.4^a^	Bernardotto et al., 2009
								0.6^b^	
Pd-Au/TiO_2_	2	37	7 O_2_, 3.5 H_2_, 89.5CO_2_	H_2_O, CH_3_OH	60	64	2.9	0.3	Edwards et al., 2005
Pd_0.1_Au_0.0333_Cs _2.5_H_0.2_PW_12_O_40_	2	40	7 O_2_, 3.5 H_2_, 89.5 CO_2_	H_2_O, CH_3_OH	–	97	29.4	0.3	Ntainjua et al., 2012
Pd-Au/C	2	37	7 O_2_, 3.5 H_2_, 89.5 CO_2_	H_2_O, CH_3_OH	98	160	6.4	1.0	Edwards et al., 2009

a *After 5 h on stream*.

b *After 12 h on stream*.

As indicated above, the minimal required H_2_O_2_ concentration produced by direct synthesis for practical applications is estimated to be 1 wt.% or 0.23 M in a methanol solution (Garcia-Serna et al., 2014). In Edwards et al. (2005) and Ntainjua et al. (2012) the maximum reached H_2_O_2_ concentration was *ca*. 0.3 wt.% and was limited by the reaction of H_2_O_2_ hydrogenation. The perfectly selective Pd-Au/C catalyst allowed to reach H_2_O_2_ concentration of more than 1.0 wt.% by several gas top-ups in a closed batch system at 37 bar pressure and 2°C (Edwards et al., 2009). The closer conditions of atmospheric pressure and flowing gas feed were utilized in Bernardotto et al. (2009) and H_2_O_2_ concentrations of 0.4 and 0.6 wt.% were reached after 5 and 12 h on stream, respectively. Thus, the target of 1.0 wt.% was likely achievable. In the present work the maximum H_2_O_2_ concentration reached is 0.1 wt.% after 5 h on stream, which is less than that of the best catalysts. The estimation of H_2_O_2_ synthesis and hydrogenation rates for PdAu_2_/PSi catalyst gives the maximum achievable H_2_O_2_ concentration at the level of *ca*. 0.8 wt.%, which could be reached by increasing reaction time and/or catalyst loading.

Thus, it is clear, that further catalyst improvement is required. However, even at this point of development it is clear, that PSi supported bimetallic Pd-Au system is a promising system to be used in the continuous flow systems for direct H_2_O_2_ synthesis. Applying the electrochemical etching technique, a layer of porous silicon could be fabricated directly on the silicon microchannel's surface and used as a support (Drott et al., 1997) for Pd-Au catalyst, avoiding the need of laborious catalyst deposition procedure. If so, silicon microreactors with a porous silicon layer onto the channel walls with deposited Pd-Au nanoparticles is a very intriguing option for performing H_2_O_2_ direct synthesis due to enhanced control abilities (Srinivas et al., 2004; Huang et al., 2014), possibility of safe operation at high temperatures and pressures (Divins et al., 2011) and wide range of reactor geometries available.

## Conclusions

In conclusion, we would like to point out key features of DCS application as a precursor and porous silicon as a support for direct H_2_O_2_ synthesis catalysts:

- The DCS [Pd(NH_3_)_4_][AuCl_4_]_2_ reduction inside the pore space of the support promotes the selective formation of Pd-Au alloy nanoparticles under very mild conditions.
- The composition of formed supported bimetallic Pd-Au nanoparticles is close to the stoichiometry of DCS.
- The Pd-Au catalysts prepared by DCS [Pd(NH_3_)_4_][AuCl_4_]_2_ reduction is active in direct H_2_O_2_ synthesis.
- PSi is porous material that could effectively enhance dispersion of supported metal nanoparticles.
- PSi with partially oxidized surface is an inert material with respect to undesirable H_2_O_2_ decomposition and hydrogenation reactions and thus could be used as a catalysts support for direct H_2_O_2_ synthesis reaction.
- The surface of PSi-supported catalysts is coated by Si^0^ and SiO_2_ sites and thus PSi could be easily wetted by different solvents, such as water, methanol, ethanol, and others.
- PSi supported Pd-Au catalysts are quite active and selective in direct H_2_O_2_ synthesis.
- PSi's surface H-terminal atoms could effectively reduce different Pd and Au precursors with the formation of bimetallic Pd-Au alloy nanoparticles. Thus bimetallic Pd-Au nanoparticles could be easily deposited onto already established PSi layer.
- PSi is a promising support for direct hydrogen peroxide synthesis from engineering point of view due to its high thermal stability and conductivity, possibility of safe operation at high temperatures and pressures, and well-established silicon processing techniques, providing the possibility to design reactors of complex internal geometry with surface porous silicon layers.

## Author contributions

DP: catalytic experiments, overall results analysis; DM: catalytic experiments; KL: FTIR experiments; PS: catalyst preparation, BET analysis; YS: XRD analysis; PP: precursors preparation, ICP AES analysis; DS: XPS analysis; VS: results analysis; AL: concept of PSi-supported catalysts, supervision of catalyst testing, and manuscript preparation and editing.

### Conflict of interest statement

The authors declare that the research was conducted in the absence of any commercial or financial relationships that could be construed as a potential conflict of interest. The reviewer, YZ, and handling Editor declared their shared affiliation.
